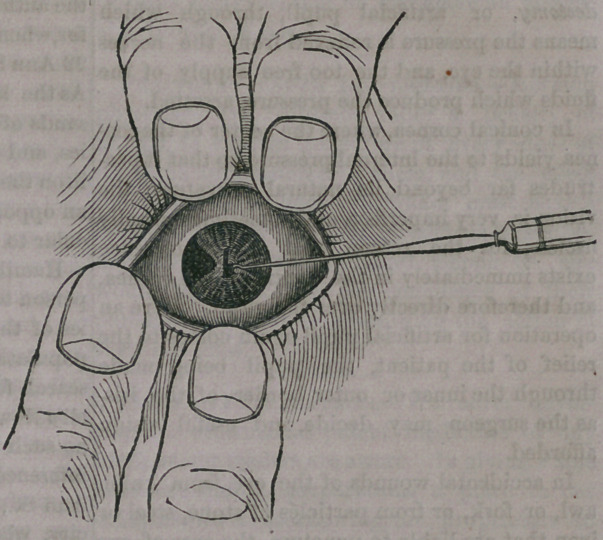# Artificial Pupil

**Published:** 1871-04

**Authors:** 


### ARTIFICIAL PUPIL.

By an artificial pupil we do not mean, as is generally supposed, that some artificial, optical arrangement is thrust into the eye, and there allowed to remain, with a view of affording vision to a blind eye; nor that an artificial, or "glass eye," is inserted to cover the deformity occasioned by the loss of the natural eye; an artificial pupil is an entirely different affair, as we shall attempt to explain.

If our readers have carefully followed us in our descriptions of the anatomy of the eye, which we have given them in this series of articles, they are prepared to understand what we mean when we speak of the *iris* and *cornea*, the two portions of the eye most concerned in what we are about to describe. It will be remembered that the cornea is the front and clear portion of the eye, and that the iris is the curtain immediately back of it, which designates the color of the eye, and through which is the little, round, black opening, called the pupil. Well, when this cornea becomes opaque,—has upon its surface what is ordinarily called a "film" or "pearl," (which we described in our last article); or when the pupil has become obstructed by a deposit of a whitish or gray matter, called lymph, which occurs in inflammation of the iris; or when the iris has become adhered to an ulcer in the cornea, distorting or closing the pupil; this operation, for an artificial pupil, comes to the relief of the patient, and affords useful vision to an eye that was before blind.

In order to illustrate the operation, let us suppose that we have a patient whose left eye is covered with a dense opacity, extending over nearly two-thirds of the cornea, and that there exists a clear portion of the cornea on the side next the nose, but, the pupil being covered by the "film," no light can pass through it. We now take a very sharp, spear-shaped instrument, and force it through the opaque spot into the chamber occupied by the iris. We then seize the iris, near its margin, with a delicate hook, and carefully and slowly withdrawing the instrument bring the iris with it, by tearing it loose from its outer margin, until the opening through it is as large as may be desired. We then have an *artificial pupil*, that is, an artificial opening through the iris, for the passage of light.

The pupil will then present a triangular appearance, as represented below:

Of course, an artificial pupil is not always made in the same locality, by the same means, or for the same purpose. There are several plans for making the pupil, but the one above described, will answer the purpose of an explanation as to what such an operation really is.

There exist quite a number of diseases of the eye, wherein it becomes necessary to remove pieces from the iris, either to relieve pain or to restore vision.

In ulcerations of the cornea, where we not only have opacity, but also an adhesion of the iris to the ulcer, producing considerable pain and preventing the healing of the ulcer, *iridectomy*, or cutting out a portion of the iris, is performed, giving instant relief from pain, and permitting the ulcer to heal. The operation is also made to relieve pressure upon the retina, in the disease known as *glaucoma*. This disease attacks the patient with a sharp, acute pain in the globe of the eye, accompanied with gradual loss of vision. The pupil presents a greenish-yellow appearance, is slightly dilated, and responds feebly when exposed to a brilliant light. Objects appear as though seen through a fog—indeed, the symptoms are nearly the same as those we described in our last article, as being peculiar to *cataract*, save that in cataract pain is rarely, if ever, experienced, while in *glaucoma* it is always severe in the eyeball, forehead, and temples, while the eye seems full, swollen, and tender to the touch, the result of the swollen condition of the humors inside. All this pain, and dullness of vision, can be *instantly* arrested by means of this wonderful operation of *iridectomy*, or artificial pupil, through which means the pressure is relieved from the nerves within the eye, and the too free supply of the fluids which produce the pressure, arrested.

In conical cornea, where the center of the cornea yields to the internal pressure, so that it protrudes far beyond its natural curvature, the vision is very imperfect, and sometimes wholly useless, for the reason that the prominence exists immediately in the centre of the cornea, and therefore directly over the pupil. Here an operation for artificial pupil again comes to the relief of the patient, the pupil being made through the inner or outer border of the iris, as the surgeon may decide, and useful vision afforded.

In accidental wounds of the eye, from knife, awl, or fork, or from particles of stone, steel or iron that are liable to puncture the eyes of our artizans, the lens is apt to be injured, which swells until it comes in contact with the iris, producing excrutiating pain. So great is the pain from this cause, that the patient imagines that some foreign substance has found lodgement in the eye, and is responsible for his suffering. If, in such cases, the sufferer applies to the skillful ophthalmic surgeon, permanent and *immediate* relief is afforded through the operation for artificial pupil.

In inflammation of the iris, called *iritis*, when all other means of treatment fail in arresting the disease and consequent pain, the difficulty is at once controlled by removing a portion of the iris. Indeed, the benefits derived from this comparatively modern operation, are beyond estimate. It not only gives sight to the blind, but is the means of curing many diseases of the eye,and arresting the most excrutiating of pains, that aforetime, were considered beyond the control of the ophthalmic surgeon.

## Figures and Tables

**Figure f1:**